# Principal Component Analysis as a Statistical Tool for Concrete Mix Design

**DOI:** 10.3390/ma14102668

**Published:** 2021-05-19

**Authors:** Janusz Kobaka

**Affiliations:** Faculty of Geoengineering, University of Warmia and Mazury in Olsztyn, 10-720 Olsztyn, Poland; janusz.kobaka@uwm.edu.pl

**Keywords:** concrete mix design, PCA, compressive strength, splitting tensile strength, consistency

## Abstract

With the recent and rapid development of concrete technologies and the ever-increasing use of concrete, adapting concrete to the specific needs and applications of civil engineering is necessary. Due to economic considerations and care for the natural environment, improving the methods currently used in concrete design is also necessary. In this study, the author used principal component analysis as a statistical tool in the concrete mix design process. Using a combination of PCA variables and 2D and 3D factors has made it possible to refine concrete recipes. Thirty-eight concrete mixes of different aggregate grades were analyzed using this method. The applied statistical analysis showed many interesting relationships between the properties of concrete and the content of its components such as the clustering of certain properties, showing dependence between the properties and the quantities of certain ingredients in concrete, and reducing noise in the data, which most importantly simplifies interpretation. This method of analysis can be used as an aid for concrete mix design.

## 1. Introduction

With the progression of civilization, a primary concern in civil engineering is building modern infrastructures for the industry and human housing needs. Concrete is still a commonly used material in construction all over the world [1,2,3,4], with its use in many applications and a variety of compositions and production technologies [5]. The concrete industry consumes the second greatest amount of natural resources [6]; thus, proper concrete design is important for environmental [7,8] and economic reasons [9,10]. Decisive initiatives should be taken today towards optimizing mix designs by taking into account its environmental impact such that the use of natural resources can be reduced [7]. Concrete mix design is a complex process, and to achieve concrete with desirable properties, many methods have been developed. Nowadays, various types of by-products, such as fly ash, silica fume, and rice husk ash, have been widely used as pozzolanic materials in concrete [11]. Additionally, chemical admixtures are essential materials and the core technology for manufacturing modern concrete in high-tech fields [12]. However, the more components there are in concrete, the more complex the design process becomes. The difference between poor-quality and good-quality concrete rests not so much on the choice of ingredients but mainly on the proportions [13]. In 1968, Powers [14] noticed that, at the macro-scale, successive filling of voids by smaller particles can increase the packing density of the aggregate [15]. Increasing the packing densities of the aggregate and cementitious materials allows the manufacturer to produce a high-performance concrete [15,16]. The most popular are methods derived from the three equations method [17,18], which allows a user to design concrete characterized by well-packed ingredients. Currently, the most popular mix design methods are the maximum density method, the fineness modulus method, the American Concrete Institute (ACI) mix design method, the Road Research Laboratory (RRL) method, and the Department of Energy (DOE) method [19]. There have been also some efforts to develop computer-aided approaches for mix design, such as an artificial neural network (ANN)-based method [11,20].

Principal component analysis (PCA) is a powerful tool that finds internal correlations within a set of data and develops a statistical representation of these datasets [21]. Moreover, it is central to the study of multivariate data [22]. In PCA, a set of factor axes in n-dimensional space is created by a rotation of the original set describing multidimensional objects in an attempt to achieve a simple structure [23]. The zero value in factor axes is the focal point represented by mean values of all variables. The main goals of PCA are to identify hidden patterns in a data set, to reduce the dimensionality of the data by removing the noise and redundancy in the data, and to identify correlated variables [24]. PCA has gained popularity by showing strong patterns especially in complex datasets [25]. The areas of application of PCA include biology [26,27], medicine [28,29], pharmacy [30], climatology [31], civil engineering [32,33], and many others. There were also some attempts to use PCA in concrete mix design; e.g., Deepika [34] used PCA variables to improve concrete mix design, while Boukhatem [35] used them to predict concrete properties. In this paper, the author proposes using a combination of PCA variables and 2D and 3D factors to refine the concrete design process.

## 2. Materials Used, Preparation of Specimens, and Testing Methods

The data used for the analysis are based on the author’s previous test results [36]. The concrete mixes used in the tests consisted of Portland Cement CEM I 32.5N manufactured in Kujawy cement plant located in Bielawy, Poland; three fractions of the aggregate, namely 0–0.5 mm, 0.5–2 mm, and 2–4 mm; and tap water (see Table 1). No additives were applied to the concrete to achieve test results based mainly on the influence of the aggregate graining on the concrete properties. The tested points from the experimental plan were plotted using three-dimensional coordinates [37] in relation to the percentage of specific fractions.

**Figure 1:**
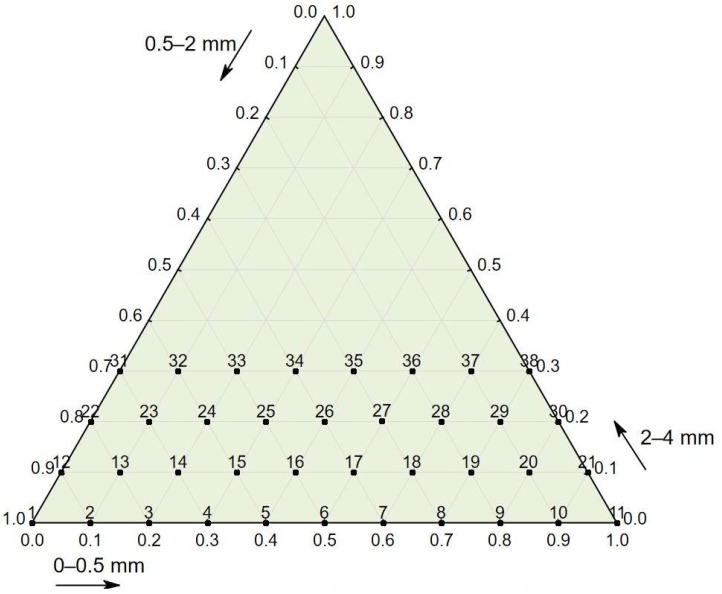
Plan of the experiment: percentage of three aggregate fractions: 0–0.5 mm, 0.5–2 mm, and 2–4 mm for 38 types of tested concrete mixes.

The aggregate fractions 0–0.5 mm and 0.5–2 mm were assessed within a scale from 0 to 100%, with steps equal to 10%, and the fraction 2–4 mm was assessed within a scale from 0 to 30%, with the same steps (see Figure 1). The water-to-cement ratio was constant and equal to 0.53 for all 38 mixes. All of the components were mixed in a concrete mixer for 2 min starting from the moment the dosing process of the ingredients ended. During molding, the concrete was compacted for 1.5 min using a vibration table characterized by 50 Hz frequency. The concrete specimens were in the form of cubes that were 150 × 150 × 150 mm. Afterward, the specimens were cured for 28 days in laboratory conditions at a temperature of +20 °C and a relative humidity of over 90%.

The research program was divided into two stages. During the first stage, the properties of fresh mixes, such as consistency, apparent density, and air content, were tested. During the second stage, the properties of the hardened concrete, namely density, compressive strength, and splitting tensile strength, were examined. The test procedures were based on European standards (see Table 2).

## 3. Test Results, Analysis, and Discussion

The test results of the fresh concrete mix (see Table 3) showed that its consistency ranged from 4.5 s, which characterizes consistency V4, to 9.2 s, which characterizes consistency V3, according to the EN 206 standard. The apparent density ranged from 2090 to 2280 kg/m^3^, and the air content ranged from 2.5 to 9.0%.

**Figure 2:**
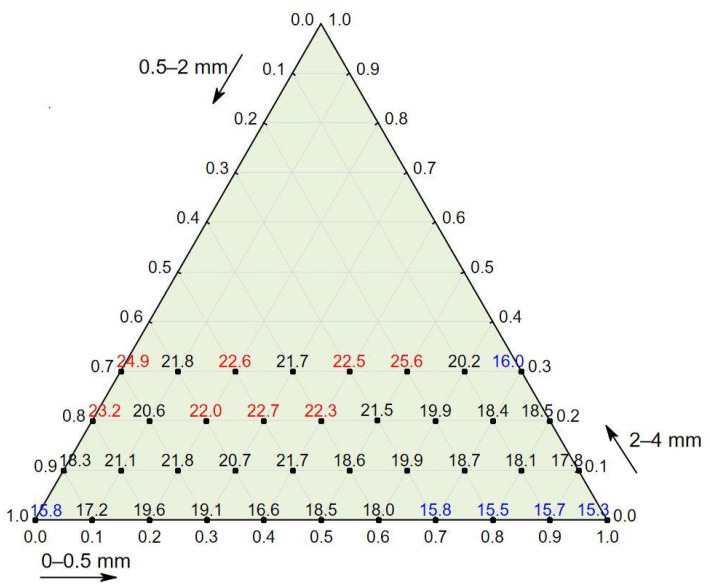
Compressive strength of concrete in relation to the percentage of three aggregate fraction groups. Red represents a compressive strength of 22 MPa or above, and blue represents a strength of 16 MPa or below.

**Figure 3:**
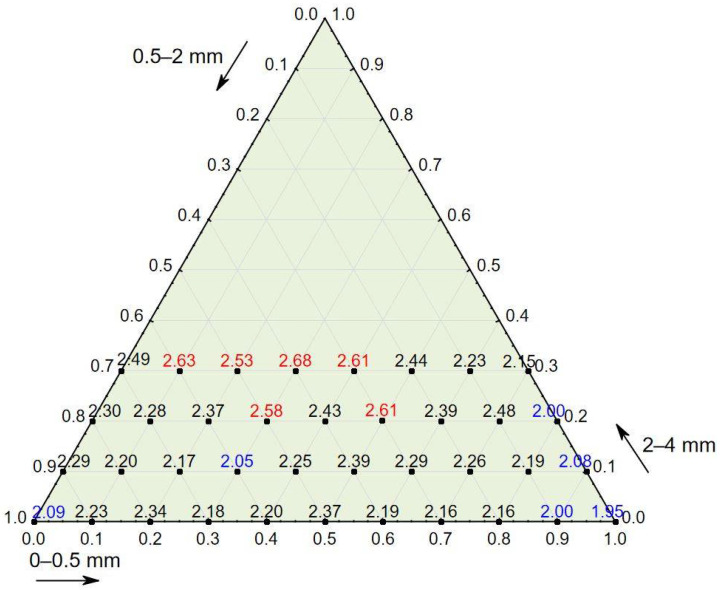
Splitting tensile strength of concrete in relation to the percentage of three aggregate fraction groups. Red represents a strength over 2.5 MPa, and blue represents a strength below 2.10 MPa.

The test results for concrete in a hardened state showed that the apparent density ranged from 1996 to 2217 kg/m^3^, that the compressive strength ranged from 15.30 to 25.60 MPa, and that the splitting tensile strength ranged from 1.9 to 2.7 MPa (see Table 4). The compressive strength in relation to the percentage of the three aggregate fraction groups (see Figure 2) shows that concrete characterized by the highest values of compressive strength also contained the most aggregate, 2–4 mm (up to 30%), and that concrete characterized by the lowest values contained the finest aggregate, 0–0.5 mm (up to 50%); this also applied to splitting tensile strength (see Figure 3).

**Figure 4:**
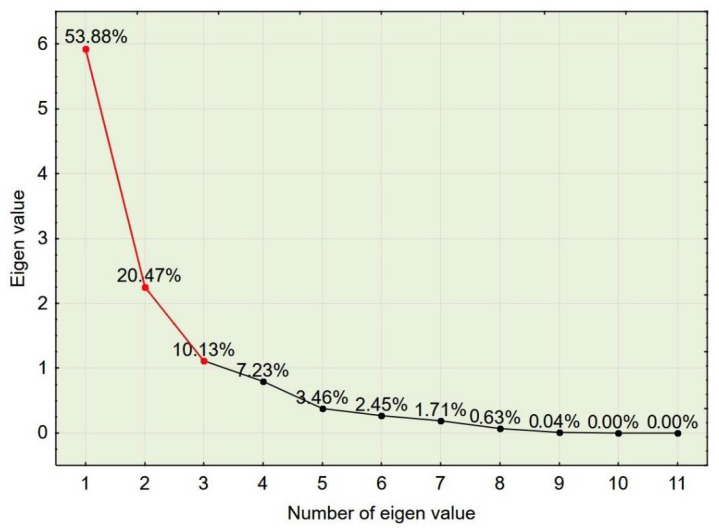
Scree plot of eigenvalues.

In order to determine the number of factors used in PCA [38], a scree plot of eigenvalues was constructed. One can see that the “elbow” of the graph where the eigenvalues appear to level off is found at eigenvalue 3, which means that factors to the left of this point should be retained as they are significant. The first two factors explain 74.35% of the variance, while the first three factors explain 84.47% of the variance (see Figure 4). Two or three factors can be visualized in 2D or 3D plots.

In the PCA analysis (see Table 5), the variables taken into account were concrete ingredients (designated as 1 to 5), the properties of the fresh concrete mix (designated as 6 to 8), and the properties of the hardened concrete (designated as 9 to 11). The variables characterized by the highest contributions of the three factors are marked with red in the table: in factor 1, they were cement, water content, and concrete density; in factor 2, they were aggregates 0–0.5 mm and 0.5–2 mm and air content; and in factor 3, they were consistency, aggregate 0.5–2 mm, and air content.

**Figure 5:**
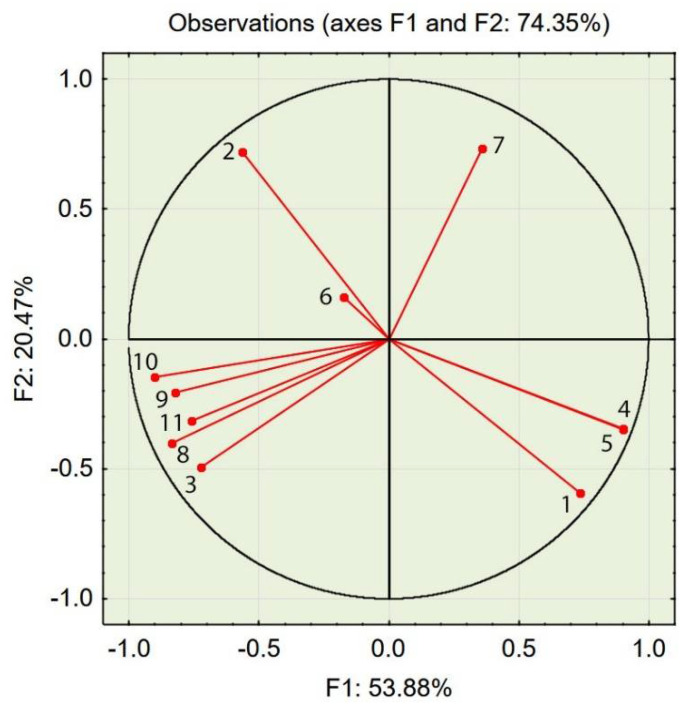
PCA projection of variables set in a 2D factor loading space (for the variable designations, see Table 5).

In the PCA projection of the variables set in the 2D factor loading space (see Figure 5), one can see that variables 4 and 5 (cement and water content, see Table 5) were plotted along the same direction, which is justified because the water/cement ratio was equal for all concrete mixes in the experiment; thus, those variables are strongly correlated.

Placing variables 4 and 5 in the same direction is an example of reducing the noise of the data using PCA. Variables 8, 9, and 10 (mix density, compressive strength, and concrete density, respectively) are strongly correlated with each other because their projections lie close to each other. These variables are also strongly correlated with variable 3 (aggregate 2–4 mm), which indicates that a high content of this aggregate is correlated with high densities of the fresh mix and the hardened concrete and high compressive strengths. Variable 7 (air content in the fresh mix) is almost directly located on the side opposite to variable 3, which means that a high content of the coarsest fraction (aggregate 2–4 mm) is correlated with low values of air content in the fresh concrete mix.

**Figure 6:**
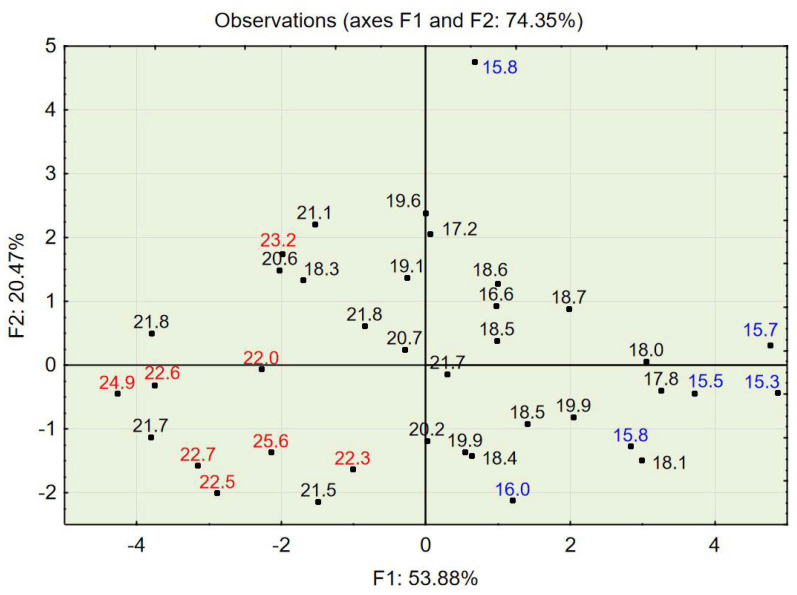
PCA with object grouping in a two-dimensional space on the basis of concrete composition in relation to concrete properties. Compressive strength: red represents a strength of 22 MPa or above, and blue represents a strength of 16 MPa or below.

**Figure 7:**
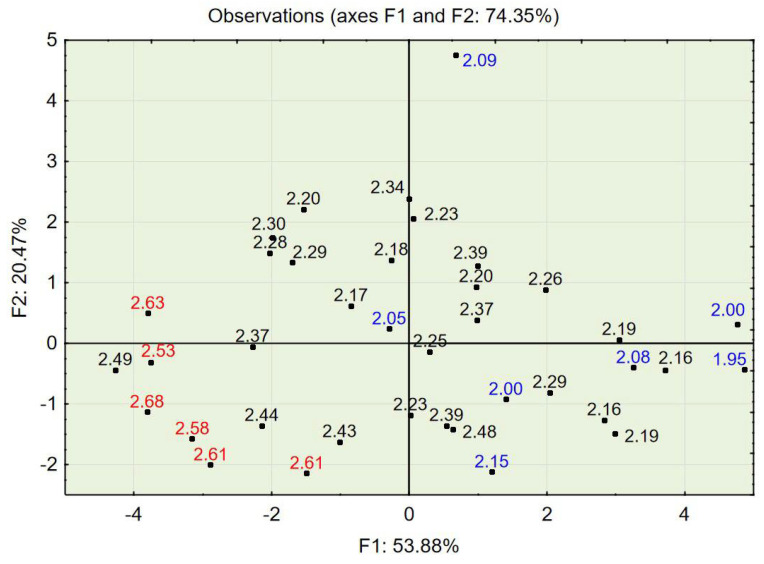
PCA with object grouping in a two-dimensional space on the basis of concrete composition in relation to concrete properties. Splitting tensile strength: red represents a strength over 2.5 MPa, and blue represents a strength below 2.10 MPa.

PCA with object grouping in a two-dimensional space shows that most cases characterized by a compressive strength of 22 MPa or above (see Figure 6) and a splitting tensile strength over 2.5 MPa (see Figure 7) are located in the bottom left of the two charts. Variables 3, 8, 9, 10, and 11 (see Figure 5)—assigned to aggregate 2–4 mm, mix density, compressive strength, concrete density, and splitting tensile strength—are also located in this area of the chart. One can conclude that a high volume of the coarse aggregate is correlated with higher densities of the concrete in the fresh and hardened states and with higher compressive and splitting tensile strengths.

Most cases characterized by a compressive strength of 16 MPa or below (see Figure 6) and a splitting tensile strength over 2.5 MPa are located in the bottom right of the two charts (see Figure 7). Variables 1, 4, and 5—assigned to aggregate 0–0.5 mm, cement, and water content—are also located in this area of the chart (see Figure 5). One can conclude that a high volume of fine aggregates is correlated with higher contents of water+cement paste because of the high specific area of very fine aggregates; however, due to the constant w/c ratio, it did not improve with regard to compressive and splitting tensile strengths.

**Figure 8:**
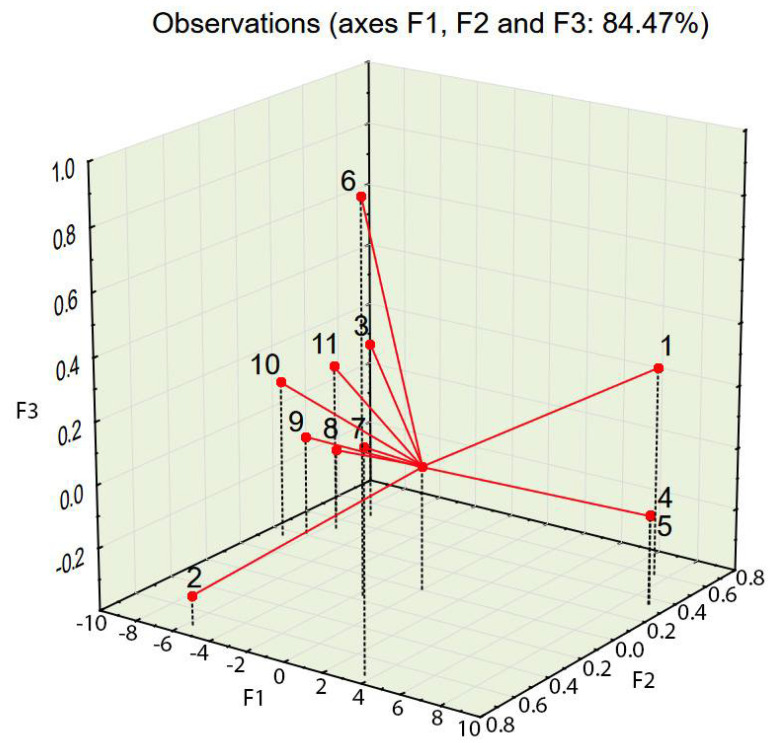
PCA projection of variables set in a 3D factor loading space (for the variable designations, see Table 5).

**Figure 9:**
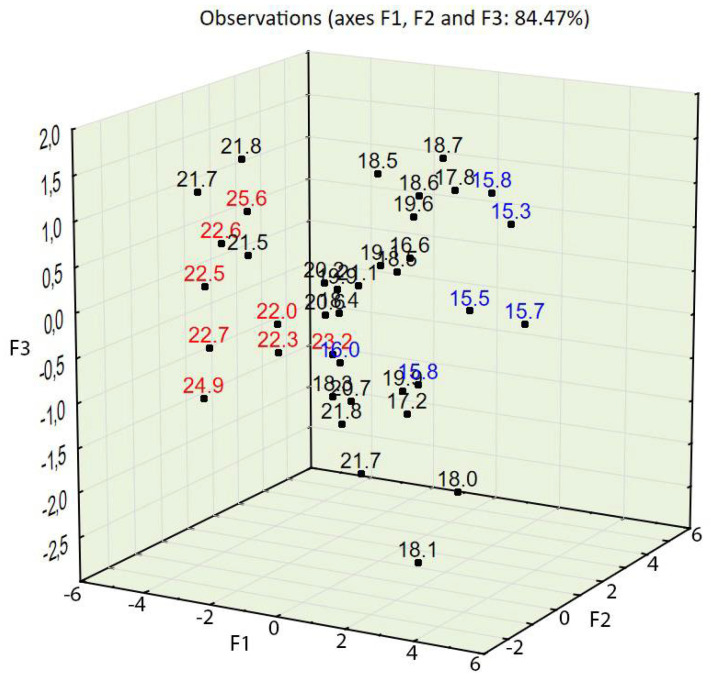
PCA with object grouping in a three-dimensional space on the basis of concrete composition in relation to concrete properties. Compressive strength: red represents a strength of 22 MPa or above, and blue represents a strength of 16 MPa or below.

**Figure 10:**
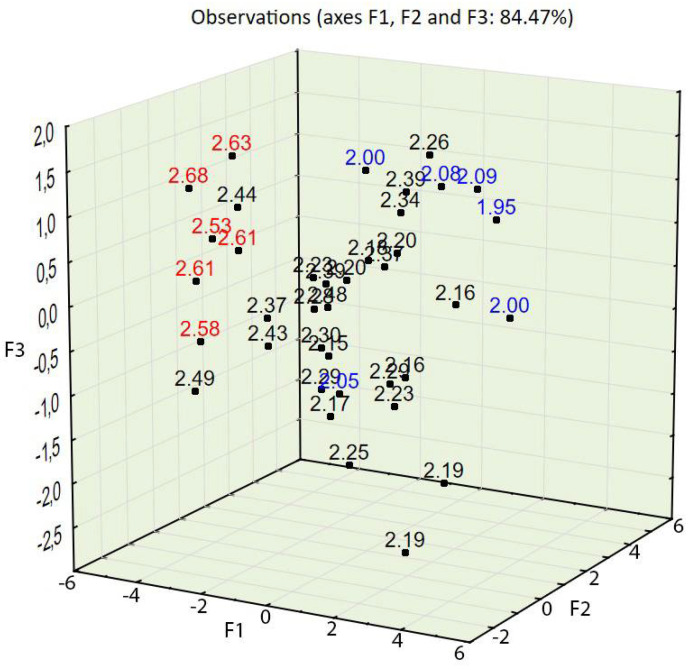
PCA with object grouping in a three-dimensional space on the basis of concrete composition in relation to properties. Splitting tensile strength: red represents a strength over 2.5 MPa, and blue represents a strength below 2.10 MPa.

Variables 8, 9, and 10—mix density, compressive strength, and concrete density in the hardened state, respectively (see Table 5)—are located at positions similar to those of the points of highest compressive and splitting tensile strengths (see Figure 8, Figure 9 and Figure 10). Variable 1—aggregate 0–0.5 mm—is located at a position on the chart similar to that of the points of lowest compressive and splitting strengths.

**Figure 11:**
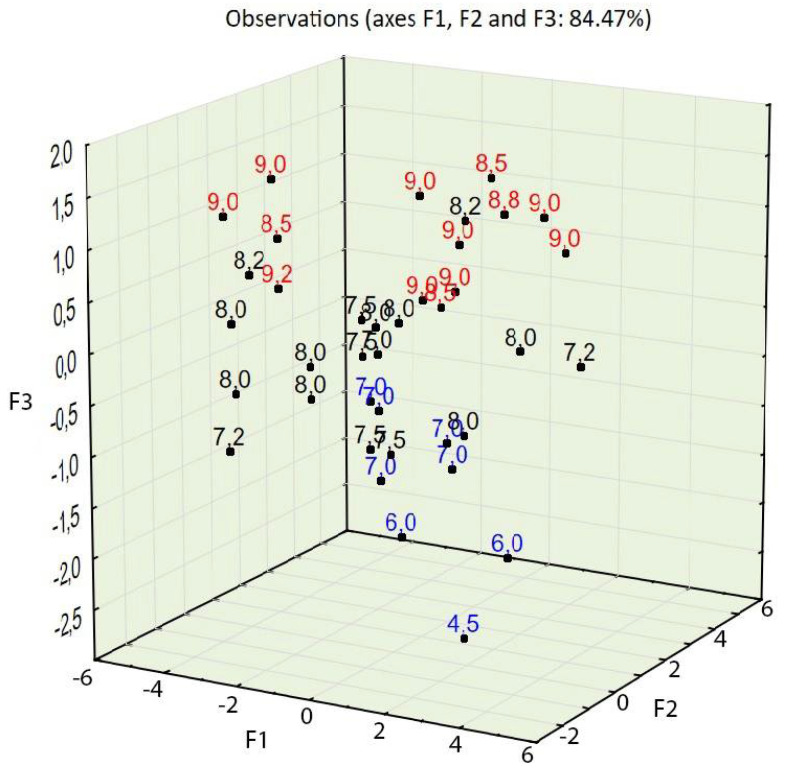
PCA with object grouping in a three-dimensional space on the basis of concrete composition in relation to properties. Consistency: red represents 8.5 s or above, and blue represents 7 s or below.

Taking into account the third factor and adding the third dimension to the 2D chart (compare Figure 5 and Figure 8) resulted in consistency being an important property of concrete, largely influencing the statistical model created using PCA. The contribution of consistency (variable 6) is high, at 66.2% (see Table 5). This phenomenon was not visible in the 2D chart (compare Figure 5 and Figure 8). In the 3D model (see Figure 8), cases characterized by consistency of 8.5 s or above were plotted at the top of the chart and cases characterized by consistency of 7 s or below were plotted at the bottom of the 3D chart (see Figure 11).

The PCA provided in the experiment described above showed a strong tendency to group cases with similar properties. The positions of cases characterized by desirable properties, i.e., high compressive strength (see Figure 6 and Figure 9), splitting tensile strength (see Figure 7 and Figure 10), or consistency (see Figure 11) are situated along the same direction as the variables that influenced the properties the most (see Figure 5 and Figure 8). A proper change in these values influences a change in the desirable properties of concrete. This is a tool useful for better understanding the concrete design process. This tool is also an excellent aid in refining the composition of a concrete mixture.

## 4. Conclusions

The principal component analysis method was used as a concrete mix design tool to obtain the following conclusions:

- Clustered cases of certain properties were grouped together; i.e., cases characterized by high compressive and splitting tensile strength were plotted together.
- A dependence between the properties and quantities of certain ingredients in concrete was observed; for instance, a high compressive strength corresponded to a high content of coarse aggregate fractions, and a low compressive strength corresponded to a high content of fine aggregate fractions.
- Noise was reduced in the data, which simplified the interpretation of most of the important factors influencing the model: due to the water/cement ratio being constant in the experiment, these variables were plotted together on the chart; other correlated variables such as mix density and concrete density were plotted close to one another.
- Elements that influenced the model to a large extent were recognized; in factor 1, they were water and cement content and concrete density.
- PCA was found to be useful as an aid for concrete mix design.
- It is also an excellent aid in refining the composition of a concrete mixture with certain properties using a combination of PCA variables and 2D and 3D factors to refine the concrete design process.
- It could also be useful for designing other types of concretes by relying on the test results of these concretes.

## Figures and Tables

**Table 1:** Composition of concrete mixes used in the experiment.

No.	Aggregate (kg/m^3^)			Cement (kg/m^3^)	Water (kg/m^3^)	No.	Aggregate (kg/m^3^)			Cement (kg/m^3^)	Water (kg/m^3^)
	0–0.5 (mm)	0.5–2 (mm)	2–4 (mm)				0–0.5 (mm)	0.5–2 (mm)	2–4 (mm)		
1	0	1570	0	358	189	20	1141	143	143	472	248
2	157	1417	0	397	209	21	1279	0	142	457	241
3	309	1235	0	394	207	22	0	1285	321	370	194
4	480	1121	0	384	202	23	167	1167	333	350	184
5	614	921	0	422	222	24	331	996	331	375	198
6	755	755	0	429	226	25	512	853	341	376	198
7	878	585	0	452	238	26	628	628	314	429	226
8	1007	432	0	488	257	27	810	491	327	410	216
9	1107	277	0	487	256	28	904	302	302	439	231
10	1225	136	0	478	251	29	1065	152	304	425	224
11	1362	0	0	492	259	30	1226	0	306	420	221
12	0	1480	164	380	200	31	0	1209	518	354	186
13	163	1303	163	360	190	32	168	1008	504	346	182
14	319	1118	160	398	210	33	344	860	516	341	180
15	479	958	160	405	213	34	522	696	522	343	181
16	617	771	154	419	221	35	670	502	502	379	200
17	751	601	150	403	212	36	817	327	490	378	199
18	850	425	142	474	249	37	948	158	474	403	212
19	1025	293	146	418	220	38	1084	0	465	431	227

**Table 2:** Composition of concrete mixes used in the experiment.

Subject of Test	Tested Property	Standard Number	Stage
Fresh concrete mix	Consistency	EN 12350-3:2001	I
	Apparent density	EN 12350-6:2011	
	Air content	EN 12350-7:2011	
Hardened concrete	Apparent density	EN 12390-7:2011	II
	Compressive strength	EN 12390-3:2011	
	Splitting tensile strength	EN 12390-6:2011	

**Table 3:** Fresh concrete mix properties—stage I of the tests.

No.	Consistency	Apparent Density	Air Content	No.	Consistency	Apparent Density	Air Content
(-)	(s)	(kg/m^3^)	(%)	(-)	(s)	(kg/m^3^)	(%)
1	9.0	2117	9.1	20	4.5	2147	5.1
2	7.0	2180	5.8	21	8.8	2119	6.5
3	9.0	2145	7.2	22	7.0	2170	6.9
4	9.0	2187	4.8	23	7.5	2201	6.2
5	9.0	2178	5.2	24	8.0	2231	4.3
6	8.5	2165	5.5	25	8.0	2280	2.5
7	6.0	2152	5.3	26	8.0	2225	3.2
8	8.0	2184	3.3	27	9.2	2262	2.6
9	8.0	2127	5.5	28	8.0	2178	4.7
10	7.2	2090	7.2	29	7.0	2170	5.4
11	9.0	2113	5.8	30	9.0	2173	5.5
12	7.5	2224	4.5	31	7.2	2267	3.6
13	8.0	2179	6.8	32	9.0	2208	6.0
14	7.0	2205	4.8	33	8.2	2241	4.9
15	7.5	2215	4.2	34	9.0	2264	3.9
16	6.0	2182	5.1	35	8.0	2253	3.4
17	8.2	2117	8	36	8.5	2211	5.1
18	7.0	2140	5.3	37	7.5	2195	5.5
19	8.5	2102	8.2	38	7.0	2207	3.8

**Table 4:** Hardened concrete properties—stage II of the tests.

No.	Apparent Density	Compressive Strength	Splitting Tensile Strength	No.	Apparent Density	Compressive Strength	Splitting Tensile Strength
(-)	(kg/m^3^)	(MPa)	(MPa)	(-)	(kg/m^3^)	(MPa)	(MPa)
1	2058	15.8	2.09	20	2080	18.1	2.19
2	2112	17.2	2.23	21	2071	17.8	2.08
3	2108	19.6	2.34	22	2151	23.2	2.30
4	2116	19.1	2.18	23	2141	20.6	2.28
5	2117	16.6	2.20	24	2157	22.0	2.37
6	2109	18.5	2.37	25	2173	22.7	2.58
7	1996	18.0	2.19	26	2144	22.3	2.43
8	2092	15.8	2.16	27	2111	21.5	2.61
9	2077	15.5	2.16	28	2123	19.9	2.39
10	2036	15.7	2.00	29	2136	18.4	2.48
11	2040	15.3	1.95	30	2129	18.5	2.00
12	2155	18.3	2.29	31	2178	24.9	2.49
13	2158	21.1	2.20	32	2217	21.8	2.63
14	2135	21.8	2.17	33	2200	22.6	2.53
15	2127	20.7	2.05	34	2179	21.7	2.68
16	2105	21.7	2.25	35	2184	22.5	2.61
17	2100	18.6	2.39	36	2154	25.6	2.44
18	2096	19.9	2.29	37	2118	20.2	2.23
19	2093	18.7	2.26	38	2110	16.0	2.15

**Table 5:** Contribution of the variables in PCA factors.

Variable Designation	Designation Assignment	Contribution of the Variables (%)		
		F1	F2	F3
1	aggregate 0–0.5 mm	9.2	15.7	6.5
2	aggregate 0.5–2 mm	5.4	23.1	8.3
3	aggregate 2–4 mm	8.8	10.8	2.7
4	cement	13.7	5.3	1.2
5	water	13.7	5.4	1.2
6	consistency	0.5	1.2	66.1
7	air content	2.2	23.9	8.1
8	mix density	11.7	7.2	2.6
9	compressive strength	11.4	1.9	0.5
10	concrete density	13.7	1.0	1.1
11	splitting tensile strength	9.7	4.5	1.7

## Data Availability

The data presented in this study are available upon request from the corresponding author.
